# Exploratory Velocity-Adjusted Upper-Extremity Loading Profiles in Fastball Pitching: A Secondary Analysis of OpenBiomechanics Data

**DOI:** 10.3390/sports14080338

**Published:** 2026-08-05

**Authors:** Yipu Zhang, Yue Wu, Han-Kyo Seo

**Affiliations:** Department of Sports Medicine, Shinhan University, Uijeongbu-si 11644, Republic of Korea; 20258604@o.shinhan.ac.kr (Y.Z.); 20248389@o.shinhan.ac.kr (Y.W.)

**Keywords:** baseball pitching, sports biomechanics, elbow varus moment, shoulder internal rotation moment, kinetic chain, velocity-adjusted loading, OpenBiomechanics, secondary analysis

## Abstract

**Background:** Fastballs delivered at comparable velocities may differ in upper-extremity loading, but cross-sectional profile differences do not establish within-pitcher biomechanical mechanisms. **Objective:** To construct an exploratory velocity-adjusted elbow–shoulder loading profile and descriptively compare non-definitional kinetic-chain characteristics associated with high-velocity low- and high-adjusted-load profiles. **Methods:** This cross-sectional secondary analysis included 411 fastballs from 100 pitchers in the OpenBiomechanics Project. Elbow varus and shoulder internal rotation moments were adjusted for pitch velocity, body mass, height, age, throwing side, and playing level; standardized residuals were averaged into a composite score. Sample tertiles defined high-velocity low-adjusted-load (HVLL) and high-velocity high-adjusted-load (HVHL) profiles, and pitcher-cluster-bootstrap and sensitivity analyses assessed robustness. **Results:** HVLL included 47 pitches from 17 pitchers, and HVHL included 37 pitches from 17 pitchers; only two pitchers contributed to both profiles, making the HVLL-versus-HVHL contrast almost entirely inter-individual. Pitch velocity was comparable (144.41 vs. 143.39 km/h; difference, 1.01 km/h; 95% CI, −0.97 to 2.88; *p* = 0.310). HVLL pitches showed smaller lead-knee extension change (6.61 degrees vs. 19.85 degrees; difference, −13.25 degrees; 95% CI, −20.37 to −5.58; FDR-adjusted *p* = 0.010) and lower lead-knee extension angular velocity at foot plant (30.67 vs. 232.77 degrees/s; difference, −202.10 degrees/s; 95% CI, −293.22 to −88.82; FDR-adjusted *p* = 0.010). These differences were attenuated when pitcher fixed effects were used to define loading residuals, further limiting within-pitcher interpretation. **Conclusions:** Because only two pitchers contributed to both profiles, the contrast was almost entirely inter-individual and describes between-pitcher performance–loading organization. It generates hypotheses but does not establish within-pitcher mechanisms, injury risk, or coaching targets.

## 1. Introduction

Fastball velocity is a central performance metric, but it does not fully describe the mechanical cost of pitching. Ball speed emerges from a coordinated kinetic chain involving lower-limb support, pelvis and trunk rotation, upper-extremity motion, and ball release [1,2,3,4], while the elbow and shoulder experience substantial varus and rotational moments [2,3,4,5]. Comparing pitches at similar velocities can therefore describe how performance–loading profiles differ across pitchers, but it cannot by itself identify how mechanical demand is redistributed within an individual pitcher [6,7].

Lead-leg and trunk mechanics are frequently associated with velocity and arm loading. Lead-knee extension can contribute to momentum redirection after foot plant, and pelvis-trunk sequencing can influence the timing of energy transfer toward the throwing arm [8,9,10,11,12,13,14,15]. These associations vary across pitchers and competitive levels, so cross-sectional between-pitcher differences cannot be assumed to represent modifiable within-pitcher strategies.

Raw elbow varus and shoulder internal rotation moments are affected by performance output and athlete characteristics, including body size, age, throwing side, and playing level. Simple division by body mass or height adjusts only one denominator and imposes a fixed proportional relationship; it cannot simultaneously account for several correlated covariates or allow non-proportional associations [16,17]. A multivariable residual model can instead estimate whether a pitch carries higher or lower loading than expected for its velocity and measured athlete profile.

The OpenBiomechanics Project provides public point-of-interest data spanning upper-extremity kinetics, lower-limb and trunk kinematics, ground-reaction forces, and energy-transfer variables [18,19,20,21,22]. It therefore permits a transparent secondary analysis of whether high-velocity pitches can be grouped according to relative elbow–shoulder loading and whether those groups differ in non-definitional kinetic-chain features.

The primary objective was to construct an exploratory velocity-adjusted upper-extremity loading score and descriptively compare non-definitional kinetic-chain characteristics of high-velocity low- and high-adjusted-load profiles. Elbow and shoulder moments were adjusted for pitch velocity and measured athlete characteristics, standardized, and combined; sample-derived cut-offs were then used to form contrast groups. These sample-derived profiles were used as cross-sectional contrast groups and were not intended to represent alternative movement strategies available to the same pitcher.

We examined whether the high-velocity profiles had comparable velocity but differed in selected lead-knee, trunk, and other non-definitional variables. Although pitches were analyzed as repeated observations nested within pitchers, the pitch-level residual models did not isolate within-pitcher deviations, and the resulting HVLL-versus-HVHL contrast was therefore interpreted as almost entirely inter-individual. The study was designed to generate hypotheses about between-pitcher performance–loading organization rather than demonstrate causal mechanisms, predict injury, or prescribe coaching changes. A supplementary analysis examined whether non-definitional variables jointly discriminated the predefined profiles.

## 2. Materials and Methods

### 2.1. Study Design

This was a cross-sectional secondary analysis of publicly available, anonymized baseball pitching data. The unit of analysis was the pitch, with repeated pitches nested within pitchers. The objective was to construct relative elbow–shoulder loading profiles among high-velocity fastballs and describe associated kinetic-chain features; no intervention, longitudinal follow-up, or injury-outcome analysis was performed.

**Figure 1:**
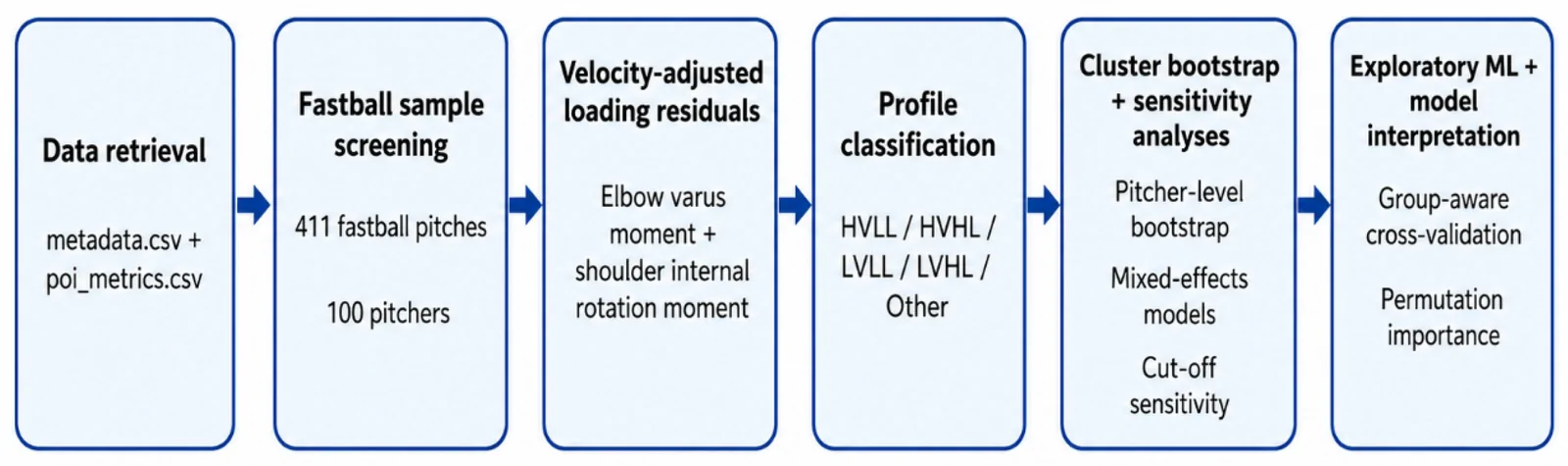
Study workflow.

The analysis proceeded in five stages. First, metadata and point-of-interest files were merged, fastballs were retained, and required variables were screened. Second, elbow varus and shoulder internal rotation moments were separately regressed on pitch velocity and measured athlete characteristics, and standardized residuals were combined into an adjusted loading score. Third, sample-derived velocity and loading cut-offs defined extreme performance–loading profiles, with HVLL versus HVHL as the primary contrast. Fourth, non-definitional kinematic variables were compared using pitcher-cluster-bootstrap, pitcher-level aggregation, mixed-effects models, and cut-off, residual-model, and PCA sensitivity analyses. Fifth, group-aware exploratory classifiers assessed multivariable profile separation without using profile-defining variables. Analyses used event-based metrics at foot plant, maximum external rotation, and ball release. The overall analytical workflow is illustrated in Figure 1.

The workflow comprised sample screening, construction of velocity-adjusted elbow and shoulder residuals, performance–loading profile classification, pitcher-aware inference and sensitivity analyses, and supplementary multivariable discrimination. POI, point of interest; HVLL, high-velocity low-adjusted-load; HVHL, high-velocity high-adjusted-load; LVLL, low-velocity low-adjusted-load; LVHL, low-velocity high-adjusted-load; ML, machine learning.

### 2.2. Data Source and Analytical Sample

The OpenBiomechanics Project baseball pitching dataset was used [18]. The analysis combined metadata.csv, which contained pitch, session, and athlete descriptors, with poi_metrics.csv, which contained throwing side, pitch type, pitch velocity, upper-extremity kinetics, lower-limb, pelvis, and trunk kinematics, centre-of-gravity variables, ground-reaction-force metrics, and energy-transfer variables.

To preserve provenance, the source files were retrieved and verified on 30 June 2026 from repository commit 8a3e889533d095a9247d70be44a861130201049a. SHA-256 checksums and source-verification instructions are included in the Zenodo reproducibility package.

The original data collection was approved by Western IRB under protocol WB-DLR-115 [18]. This secondary analysis used publicly available anonymized data and required no new recruitment or testing. Files were merged using pitch- and session-level identifiers, records coded as fastballs were retained, and complete data were required for pitch velocity, elbow varus moment, shoulder internal rotation moment, body mass, body height, age, throwing side, and playing level. The primary sample comprised 411 fastballs from 100 pitchers; extended kinetic and energy-transfer analyses used complete-case subsets. Detailed information on the variable dictionary, sample-screening flow, and missing-data summary is provided in Supplementary Tables S1–S3, respectively.

### 2.3. Performance and Upper-Extremity Loading Variables

Pitch velocity was converted from the source field to kilometres per hour using a factor of 1.609344 and is reported in km/h throughout the manuscript, tables, and figures.

Upper-extremity loading was represented by two kinetic variables: elbow varus moment and shoulder internal rotation moment. These variables corresponded to elbow_varus_moment and shoulder_internal_rotation_moment, respectively, and were reported in Newton metres. Both variables were treated as pitch-level biomechanical indicators of upper-extremity loading. They were not treated as direct measures of injury, clinical diagnosis, tissue failure, or future injury risk.

This distinction was important because the dataset did not include longitudinal injury surveillance, previous injury history, workload exposure, fatigue state, pain status, or clinical outcomes. Therefore, elbow varus moment and shoulder internal rotation moment were used only to characterize mechanical loading during pitching. No injury-prediction model was developed, and no threshold was interpreted as a safe or unsafe pitching value.

**Figure 2:**
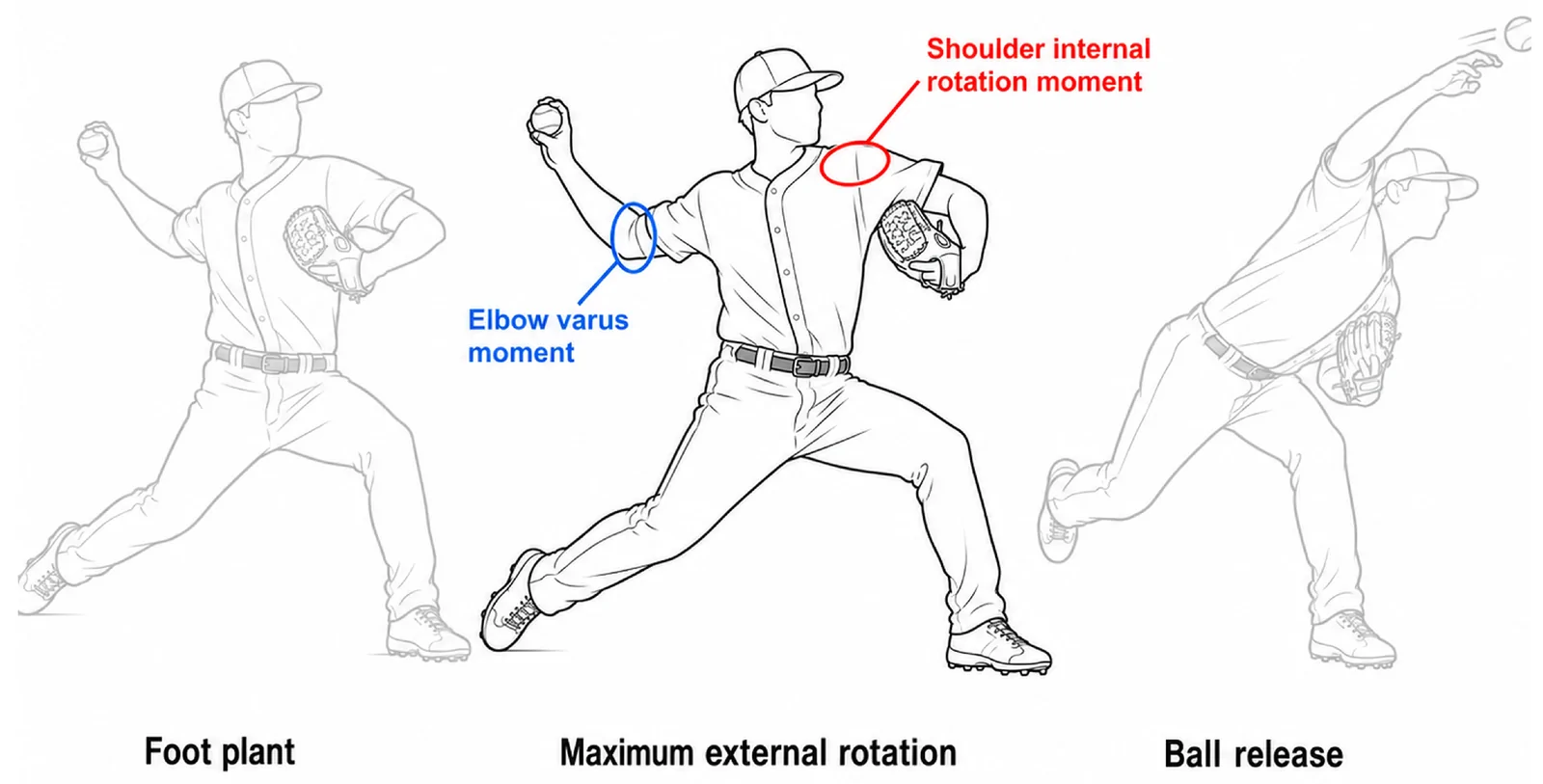
Generic schematic of the baseball pitching sequence and analyzed upper-extremity loading variables.

A generic schematic of the pitching sequence and the two upper-extremity loading regions analyzed in this study is provided in Figure 2.

The sequence illustrates foot plant, maximum shoulder external rotation, and ball release. The coloured callouts identify the shoulder internal rotation moment and elbow varus moment regions analyzed in this study. The illustration does not depict an actual study participant and does not indicate the exact timing, magnitude, or sign convention of the measured moments.

### 2.4. Kinetic-Chain Predictor Variables

Predictor variables were grouped into core kinematic variables and extended kinetic or ground-reaction-force variables. The core kinematic variables were used for the main biomechanical comparisons because they directly represented lower-limb, pelvis, trunk, stride, and whole-body motion features. These variables included stride length, stride angle, lead-knee extension angular velocity, lead-knee extension change from foot plant to ball release, maximum trunk axial rotational velocity, maximum pelvis axial rotational velocity, maximum hip–shoulder separation, trunk anterior tilt, trunk lateral tilt, trunk axial rotation, pelvis anterior tilt, pelvis lateral tilt, pelvis axial rotation, maximum horizontal centre-of-gravity velocity, and centre-of-gravity velocity at peak knee height.

Key pitching events were defined according to the OpenBiomechanics point-of-interest metrics. Foot plant was defined as the instant at which the lead foot contacted the ground, maximum external rotation was defined as the instant at which the throwing shoulder reached maximum external rotation, and ball release was defined as the instant of ball release. Lead-knee extension change represented the change in lead-knee extension angle from foot plant to ball release. Trunk and pelvis variables were extracted at the corresponding pitching events provided in the point-of-interest dataset.

Variable units followed the OpenBiomechanics data dictionary [18]. Elbow and shoulder moments were reported in Newton metres. Angular variables were reported in degrees, angular velocities in degrees per second, centre-of-gravity velocity in metres per second, ground-reaction-force variables in Newtons, and energy-transfer-related variables in Joules. Stride length was reported relative to body height and was therefore presented in body heights.

Extended kinetic and ground-reaction-force variables included lead-leg and rear-leg ground-reaction forces, hip and knee energy-transfer variables, energy generation, energy absorption, pelvis–lumbar energy transfer, and distal thorax energy-transfer variables. These variables were used as exploratory predictors and were not treated as the primary biomechanical comparison set. Variables with extreme or unstable values were screened before analysis, and complete-case analysis was used for extended variables with missing data. A complete variable dictionary, including dataset variable names, biomechanical definitions, pitching events, units, and direction interpretation, is provided in the Zenodo reproducibility package.

### 2.5. Construction of Velocity-Adjusted Upper-Extremity Loading

To compare upper-extremity loading among pitches delivered at comparable performance levels, elbow varus moment and shoulder internal rotation moment were modelled separately as pitch-level outcomes. Each outcome was adjusted for pitch velocity, body mass, body height, age, throwing side, and playing level using ordinary least squares. The throwing side contained two observed levels (right and left), with left-handed throwing as the reference category; the model therefore included one indicator for right-handed throwing. The playing level contained four observed levels (college, independent, high school, and MiLB), with college as the reference category; the model therefore included indicators for high school, independent, and MiLB. The complete design-matrix specification is reported in Supplementary Table S4 and the released analysis code.

For pitch *i*, the adjustment model for a loading variable *L* was specified as

(1) $$Li=\beta 0+\beta 1Vi+\beta 2Mi+\beta 3Hi+\beta 4Ai+\gamma TTi+\delta TPi+\varepsilon_{i}$$

where *L* denotes either elbow varus moment or shoulder internal rotation moment, *V* denotes pitch velocity, *M* denotes body mass, *H* denotes body height, *A* denotes age, *T* denotes throwing-side indicators, *P* denotes playing-level indicators, and *ε*ᵢ denotes the residual for pitch *i* from the adjustment model.The residuals from the elbow and shoulder models were standardized across the full analytical sample. The composite velocity-adjusted upper-extremity loading score was calculated as the average of the standardized elbow and shoulder residuals:

(2) $$C_{i}=\frac{Z_{i}^{elbow}+Z_{i}^{shoulder}}{2}$$

where *C* represents the composite adjusted loading score, $Z_{i}^{elbow}$ represents the standardized residual from the elbow varus moment model, and $Z_{i}^{shoulder}$ represents the standardized residual from the shoulder internal rotation moment model. Lower values of *C* indicated relatively lower upper-extremity loading after adjustment for pitch velocity and measured athlete-level characteristics, whereas higher values indicated relatively higher adjusted loading.

This residual-based approach allowed pitch velocity and multiple athlete characteristics to be adjusted simultaneously. In contrast, an unadjusted moment or simple ratio to body mass or height cannot represent several covariates at once and assumes a fixed proportional scaling relation [23]. The residuals were interpreted only as relative biomechanical loading indicators, not as causal estimates, injury-risk scores, clinical thresholds, or coaching targets.

The primary models were fitted at the pitch level and did not isolate purely within-pitcher deviations. The resulting score may therefore contain both within-pitcher and between-pitcher variation. An additional residual model with pitcher fixed effects was used to determine whether profile-associated findings persisted after accounting for pitcher-specific loading baselines.

The equal-weight mean was selected a priori as the primary composite because both residuals were standardized to the same scale, neither joint was assigned clinical priority, and a simple average preserves transparent directionality without estimating sample-specific weights. The residual correlation was examined to determine whether the two components represented a shared loading dimension.

Principal component analysis was reserved for sensitivity analysis because its weights are data-driven. The first principal component provided an alternative adjusted loading score and tested whether profile assignments and non-definitional findings depended on the transparent equal-weight definition.

Model diagnostics included coefficients, standard errors, confidence intervals, R-squared, adjusted R-squared, residual-distribution checks, influential-observation summaries, and formal collinearity assessment. Variance inflation factors were calculated from the exact dummy-coded predictor matrix used in each residual model, with the intercept excluded from interpretation. Because both outcomes and all covariates were complete for all 411 pitches, the elbow and shoulder models used the same predictor matrix and therefore yielded identical VIF estimates. Residual correlation and PCA-based sensitivity outputs were also examined. Complete diagnostics, including term-specific VIF estimates, are provided in Supplementary Table S4 and the reproducibility package. Residual diagnostic plots are shown in Supplementary Figure S1.

### 2.6. Performance–Loading Profile Classification

After construction of the composite velocity-adjusted upper-extremity loading score, pitches were classified using an exploratory two-dimensional performance–loading framework. The first dimension was pitch velocity, representing performance output. The second dimension was the composite adjusted upper-extremity loading score, representing relative upper-extremity loading after adjustment for pitch velocity and measured athlete-level characteristics.

Sample-derived tertile cut-offs defined relatively low and high values for velocity and adjusted loading. Tertiles were selected a priori to balance contrast and precision: the outer thirds produced clearly separated groups while retaining enough pitches and pitchers for cluster-based analyses. The cut-offs were not optimized against any biomechanical outcome or *p*-value and were not intended as normative, safety, injury-risk, clinical, or coaching thresholds.

This procedure produced four extreme sample-derived performance–loading profiles:

(3) $$HVLL:V_{i}\geq Q_{2/3}(V),C_{i}\leq Q_{1/3}(C)$$

(4) $$HVHL:V_{i}\geq Q_{2/3}(V),C_{i}\geq Q_{2/3}(C)$$

(5) $$LVLL:V_{i}\leq Q_{1/3}(V),C_{i}\leq Q_{1/3}(C)$$

(6) $$LVHL:V_{i}\leq Q_{1/3}(V),C_{i}\geq Q_{2/3}(C)$$

where *V* denotes pitch velocity, *C* denotes the composite adjusted upper-extremity loading score, $Q_{1/3}$ denotes the lower tertile cut-off, and $Q_{2/3}$ denotes the upper tertile cut-off. HVLL indicates high-velocity low-adjusted-load pitches, HVHL indicates high-velocity high-adjusted-load pitches, LVLL indicates low-velocity low-adjusted-load pitches, and LVHL indicates low-velocity high-adjusted-load pitches.

The primary comparison focused on the HVLL and HVHL profiles because both groups represented high-velocity fastballs but differed in relative upper-extremity loading. This comparison was intended to describe whether high-velocity pitches with relatively lower adjusted loading were associated with different non-definitional kinetic-chain features compared with high-velocity pitches with relatively higher adjusted loading. Because pitch velocity and the composite adjusted loading score were used to define profile membership, differences in these variables were treated as profile verification rather than independent biomechanical findings.

Robustness to the cut-off choice was examined using quartile definitions, which increased contrast but reduced sample size, and a median split, which increased sample size but reduced contrast. Because the primary residual score could contain between-pitcher variation, profiles were also reconstructed from a pitcher-fixed-effect residual model. Complete profile assignments and cut-off, residual-model, and PCA sensitivity analyses are provided in the reproducibility package.

### 2.7. Between-Profile Comparisons

The primary between-profile comparison was performed between the HVLL and HVHL profiles. This comparison was selected because both groups represented high-velocity fastball pitches, whereas they differed in the composite velocity-adjusted upper-extremity loading score used for profile classification. Therefore, differences in elbow varus moment, shoulder internal rotation moment, and the composite adjusted loading score were treated as profile verification rather than independent biomechanical findings. The primary inferential focus was placed on non-definitional lower-limb, pelvis, trunk, stride, and centre-of-gravity variables.

Continuous variables were summarized as means and standard deviations. Initial pitch-level comparisons were performed using Welch’s *t*-test to reduce sensitivity to unequal variances between groups [24]. Mann–Whitney U tests were also performed as non-parametric supplementary analyses [25]. Between-profile effect sizes were calculated using Hedges’ g, with negative values indicating lower values in HVLL pitches and positive values indicating higher values in HVLL pitches [26].

Multiple comparisons were controlled using the Benjamini–Hochberg false-discovery-rate procedure [27]. Core kinematic variables were treated as the primary non-definitional biomechanical comparison family, whereas extended kinetic, ground-reaction-force, and energy-transfer variables were treated as exploratory variables. False-discovery-rate correction was therefore applied separately within the core kinematic comparison family and the exploratory extended-variable family.

For transparency, the complete core kinematic comparison family included all prespecified lower-limb, pelvis, trunk, stride, and centre-of-gravity variables used in the primary non-definitional biomechanical comparison. Selected non-definitional variables central to the main-text interpretation are reported in the Results. Complete between-profile comparison results are provided in Supplementary Table S5, whereas the complete core kinematic comparison family is provided in Supplementary Table S6. False-discovery-rate adjustment was applied to the complete available family rather than only to the variables displayed in the main-text results table. Because individual pitchers could contribute multiple pitches, pitch-level significance tests were interpreted only as preliminary descriptive analyses. The primary statistical interpretation was based on pitcher-level cluster-bootstrap and sensitivity analyses. Complete pitch-level comparison results, including Welch’s *t*-test, Mann–Whitney U test, Hedges’ g, unadjusted *p*-values, false-discovery-rate-adjusted *p*-values, cluster-bootstrap estimates, and sensitivity-analysis outputs for all core and extended variables are provided in the Zenodo reproducibility package.

### 2.8. Pitcher-Level Clustering and Sensitivity Analyses

Pitch-level records were nested within pitchers, and individual pitchers could contribute more than one fastball pitch. To reduce the risk of treating repeated pitches as fully independent observations, pitcher-level cluster-bootstrap analysis was used as the primary robustness procedure for between-profile mean differences. Pitcher ID was used as the resampling unit rather than individual pitch. In each bootstrap iteration, pitchers were sampled with replacement, and all pitch records from the sampled pitchers were retained. Mean differences between HVLL and HVHL pitches were then recalculated. A total of 5000 bootstrap iterations was used to estimate 95% confidence intervals and two-sided *p*-values. Cluster-bootstrap procedures are appropriate when observations are non-independent within clusters and inference should reflect cluster-level resampling rather than simple observation-level resampling [28].

The primary cluster-bootstrap analysis retained the original tertile-based HVLL and HVHL profile labels. Tertile cut-offs were not re-estimated within each bootstrap sample. This approach evaluated the robustness of mean differences for the predefined performance–loading profiles under pitcher-level resampling.

A pitcher-level aggregated sensitivity analysis was also performed to examine whether the main findings were driven by repeated pitches from the same pitchers. In this analysis, variables were averaged within each pitcher and profile before between-profile comparisons. This analysis provided a more conservative assessment of profile differences at the pitcher level rather than the pitch level. Complete pitcher-level aggregated results are reported in Supplementary Table S7.

Mixed-effects sensitivity models were additionally fitted for the prespecified main non-definitional biomechanical outcomes [29]. These models included profile as the primary fixed-effect predictor and pitcher as a random intercept. Pitch velocity, body mass, body height, age, throwing side, and playing level were retained as covariates. Model stability was assessed by checking convergence status, the number of pitcher-level clusters, cluster-size distribution, and random-intercept variance. These models were used only as sensitivity analyses and were not interpreted as causal models. Complete mixed-effects estimates, confidence intervals, *p*-values, convergence information, cluster counts, and random-effect variance estimates are reported in Supplementary Table S8.

Cut-off sensitivity analyses were used to evaluate whether the findings depended on the tertile-based classification strategy. In addition to the primary tertile-based profiles, quartile-based and median-based profile definitions were examined. These analyses were interpreted as robustness checks rather than alternative primary definitions. Complete cut-off sensitivity results are reported in Supplementary Table S9.

Residual-model sensitivity analyses were also performed to examine whether profile classification depended on the original adjustment model. The primary residual models adjusted for pitch velocity, body mass, body height, age, throwing side, and playing level. A sensitivity model additionally accounting for pitcher fixed effects was used to evaluate whether the loading profiles and associated non-definitional biomechanical features remained consistent after accounting for between-pitcher differences. Complete residual-model sensitivity results are reported in Supplementary Table S10.

Complete cluster-bootstrap outputs, pitcher-level aggregated analyses, mixed-effects sensitivity models, cut-off sensitivity analyses, and residual-model sensitivity analyses are provided in the Zenodo reproducibility package.

### 2.9. Supplementary Exploratory Multivariable Discrimination

Supplementary exploratory multivariable discrimination analyses were conducted to examine whether non-definitional kinetic-chain variables contained multivariable signal separating the predefined HVLL and HVHL profiles. These analyses were not designed to develop an externally validated prediction model, clinical screening tool, injury-risk model, or coaching-deployment algorithm. Instead, they were used as descriptive analyses to support interpretation of between-profile biomechanical organization.

Only HVLL and HVHL pitches were included in this analysis. The binary classification label was coded as 1 for HVLL and 0 for HVHL. To avoid label leakage, variables directly used to define the profiles were excluded from all machine-learning inputs. Therefore, pitch velocity, elbow varus moment, shoulder internal rotation moment, velocity-adjusted elbow residual, velocity-adjusted shoulder residual, the primary composite velocity-adjusted loading score, and the PCA-based loading score were not included as predictors.

Two predictor sets were examined. The first predictor set included only core kinematic variables representing lower-limb, pelvis, trunk, stride, and centre-of-gravity features. The second predictor set added extended kinetic, ground-reaction-force, and energy-transfer-related variables. The expanded predictor set was treated as exploratory because these variables contained a small amount of missing data and may be more sensitive to data-processing assumptions.

Three fixed model classes were compared: L2-regularized logistic regression, random forest [30], and histogram-based gradient boosting. The models were used to describe multivariable profile separation rather than to optimize a deployable prediction system. Model implementation used scikit-learn version 1.9.0 [31]. Hyperparameter settings and predictor lists are provided in the Zenodo reproducibility package, whereas fold-level train/test distributions and class counts are reported in Supplementary Table S11.

Model evaluation used five-fold stratified group cross-validation. Stratification was used to preserve class balance between HVLL and HVHL profiles as far as possible, while pitcher ID was used as the grouping variable to prevent pitches from the same pitcher from appearing in both training and test folds. This design was selected because pitch-level records were nested within pitchers, and leakage of pitcher-specific information across training and test folds would overestimate model performance.

Model performance was summarized using the area under the receiver operating characteristic curve, accuracy, balanced accuracy, F1 score, sensitivity, and specificity. AUROC and balanced accuracy were treated as descriptive discrimination metrics rather than as evidence of external predictive validity. Because the analysis involved a small number of pitches and pitchers, model performance estimates were expected to have substantial uncertainty and were interpreted cautiously [32]. Complete model-performance summaries are reported in Supplementary Table S12, with full fold-level outputs and exploratory model-interpretation results provided in the Zenodo reproducibility package.

### 2.10. Model Interpretation

Model-interpretation analyses were conducted as supplementary exploratory analyses to describe which variables contributed to multivariable discrimination between the predefined HVLL and HVHL profiles. These analyses were used only to support interpretation of exploratory models and were not treated as primary biomechanical evidence, causal mechanisms, modifiable technical determinants, injury-risk indicators, or external prediction-validation results.

Permutation importance was calculated by measuring the reduction in the area under the receiver operating characteristic curve after randomly permuting each predictor variable. Larger reductions indicated greater contribution of that variable to model discrimination. Permutation importance was used to describe how the model separated the predefined profiles, not to estimate causal biomechanical effects.

Standardized logistic regression coefficients were also examined as a supplementary directional interpretation. Continuous predictors were standardized before model fitting. Coefficients were interpreted only as descriptive associations with the predefined HVLL label within the selected predictor set.

Because machine-learning interpretation methods can be unstable in small datasets and may reflect correlations among predictors rather than independent biomechanical effects, feature-importance results were interpreted cautiously [33]. Complete permutation-importance rankings and standardized logistic regression coefficients are provided in the Zenodo reproducibility package.

### 2.11. Software and Reproducibility

Data processing, statistical analysis, machine-learning modelling, and figure generation were performed in Python using pandas, NumPy, SciPy, statsmodels, scikit-learn, and matplotlib. The analysis was conducted using Python 3.11.9, NumPy 2.4.6, pandas 3.0.3, SciPy 1.17.1, statsmodels 0.14.6, scikit-learn 1.9.0, and matplotlib 3.11.0. The exact software environment is documented in requirements_pinned.txt and software_environment_summary.txt in the Zenodo reproducibility package.

The reproducibility package includes analysis scripts, derived datasets, velocity-adjusted loading scores, performance–loading profile classifications, statistical outputs, sensitivity analyses, machine-learning outputs, supplementary tables, supplementary figures, and figure-generation files. The original OpenBiomechanics data are not redistributed in the package; instead, the package provides scripts and derived outputs that support transparent verification of the present analyses from the original public data source.

## 3. Results

### 3.1. Analytical Sample

The final analytical sample included 411 fastball pitches from 100 pitchers. All included pitches were coded as fastballs. Most pitches were thrown by right-handed pitchers (328 pitches, 79.8%), whereas left-handed pitchers contributed 83 pitches (20.2%). The sample was mainly composed of college-level pitchers (314 pitches, 76.4%), with the remaining pitches from independent, high school, and Minor League Baseball pitchers.

Across all fastballs, mean pitch velocity was 136.31 ± 7.60 km/h. Mean elbow varus moment was 111.27 ± 19.65 Nm, and mean shoulder internal rotation moment was 106.12 ± 18.72 Nm. Mean body mass was 90.29 ± 9.90 kg, mean body height was 1.85 ± 0.07 m, and mean age was 21.29 ± 2.25 years. Core kinetic-chain variables also showed between-pitch variability. Sample characteristics are summarized in Table 1, with complete descriptive and missing-data outputs in the reproducibility package.

### 3.2. Velocity-Adjusted Upper-Extremity Loading Profiles

The elbow and shoulder adjustment models explained moderate proportions of loading variability. The elbow model had R-squared = 0.512, adjusted R-squared = 0.502, and RMSE = 13.71 Nm; the shoulder model had R-squared = 0.559, adjusted R-squared = 0.550, and RMSE = 12.41 Nm. Variance inflation factors ranged from 1.09 to 2.94 in both models. Age had the highest VIF (2.94), whereas all remaining predictors had VIFs of 2.01 or lower. These results provided no evidence of substantial multicollinearity in the fitted adjustment models.

The standardized elbow and shoulder residuals were highly correlated (Pearson r = 0.961), supporting interpretation as a shared adjusted upper-extremity loading dimension. The first principal component explained 98.05% of residual-loading variance, had equal elbow and shoulder loadings (0.707 and 0.707), and was perfectly correlated with the equal-weight score to three decimals. PCA-based profile assignments were identical to the primary assignments.

The sample-derived high-velocity cut-off was ≥140.33 km/h, and the low-velocity cut-off was ≤133.41 km/h. The lower and upper tertiles of the composite adjusted loading score were −0.447 and 0.355. These cut-offs created exploratory contrast groups only and were not normative, safety, injury-risk, clinical, or coaching thresholds.

This classification yielded 47 high-velocity low-adjusted-load pitches (HVLL; 11.4% of all pitches) from 17 pitchers and 37 high-velocity high-adjusted-load pitches (HVHL; 9.0%) from 17 pitchers. The low-velocity low-adjusted-load profile included 48 pitches from 16 pitchers, and the low-velocity high-adjusted-load profile included 50 pitches from 15 pitchers. The remaining 229 pitches were not classified into an extreme performance–loading profile.

**Figure 3:**
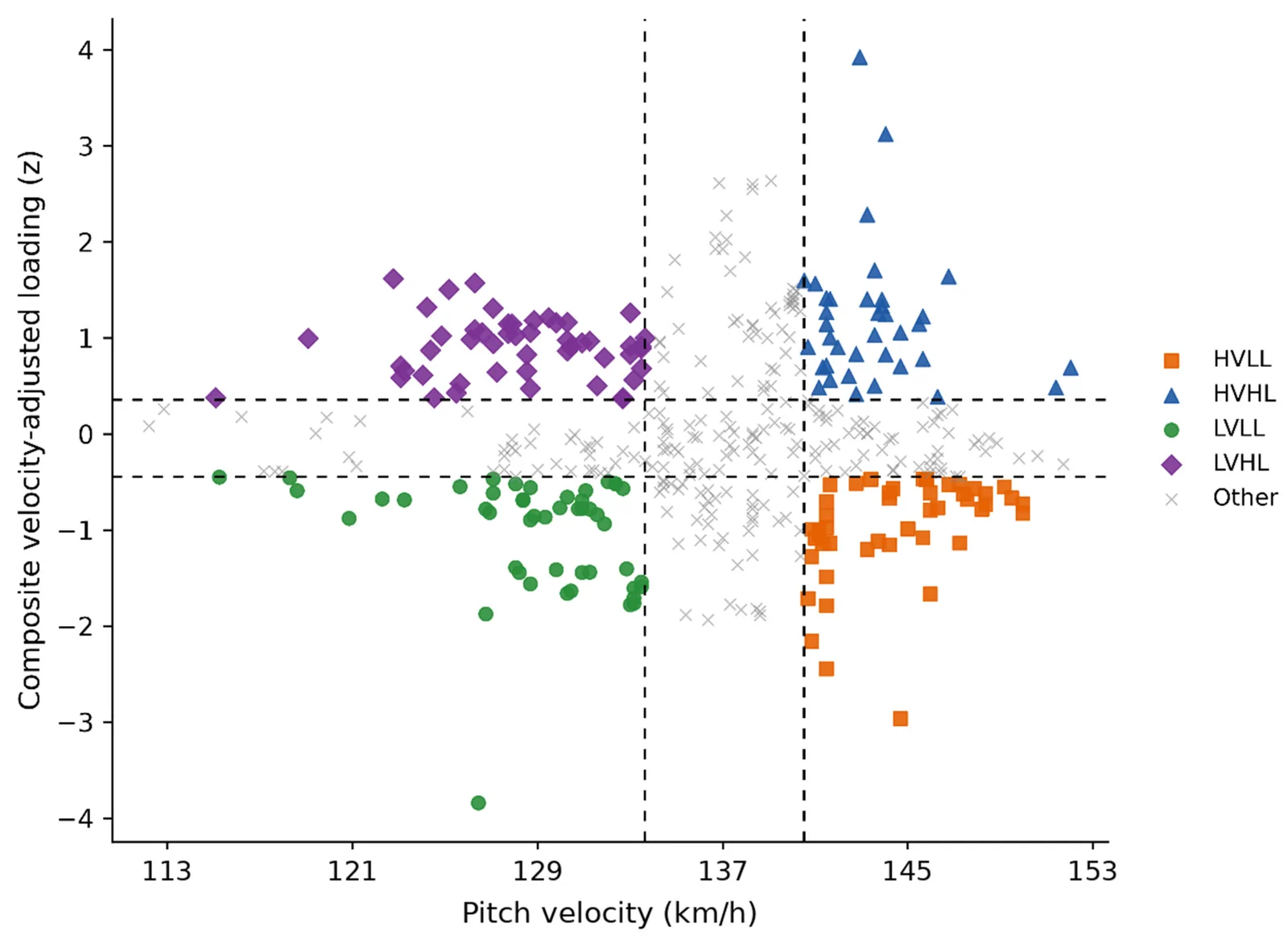
Sample-derived performance–loading profiles.

Only two pitchers contributed pitches to both the HVLL and HVHL profiles. Accordingly, the primary comparison mainly contrasted profiles generated by different pitchers rather than alternative strategies performed by the same pitchers. Pitcher-fixed-effect residual sensitivity analysis was used to examine this limitation directly. The sample-derived performance–loading profiles are illustrated in Figure 3. The corresponding profile definitions and sample sizes are summarized in Table 2.

Profiles were based on pitch velocity and the composite velocity-adjusted upper-extremity loading score. Dashed vertical lines indicate the lower and upper sample tertiles for pitch velocity (133.41 and 140.33 km/h); dashed horizontal lines indicate the loading tertiles (−0.447 and 0.355). Marker colours and shapes indicate profile classification. The cut-offs are sample-specific exploratory definitions, not normative, clinical, safety, injury-risk, or coaching thresholds. HVLL, high-velocity low-adjusted-load; HVHL, high-velocity high-adjusted-load; LVLL, low-velocity low-adjusted-load; LVHL, low-velocity high-adjusted-load.

Profiles were defined from sample tertiles of pitch velocity and composite adjusted loading. High velocity was ≥140.33 km/h and low velocity was ≤133.41 km/h. Low adjusted loading was ≤−0.447 and high adjusted loading was ≥0.355.

These values were used only to create exploratory contrast groups within the present analytical sample and should not be interpreted as normative thresholds, safety thresholds, injury-risk cut-offs, clinical thresholds, or coaching targets. Only two pitchers contributed pitches to both the HVLL and HVHL profiles; therefore, the primary high-velocity profile comparison was interpreted mainly as a between-profile comparison with substantial between-pitcher contribution. HVLL, high-velocity low-adjusted-load; HVHL, high-velocity high-adjusted-load; LVLL, low-velocity low-adjusted-load; LVHL, low-velocity high-adjusted-load.

### 3.3. Main HVLL Versus HVHL Differences

Pitch velocity was comparable between the high-velocity profiles: 144.41 km/h in HVLL and 143.39 km/h in HVHL. The pitcher–cluster-bootstrap difference was 1.01 km/h (95% CI, −0.97 to 2.88; *p* = 0.310), indicating that the lower adjusted-loading profile was not explained by lower velocity.

As expected from the profile construction, HVLL pitches showed lower upper-extremity loading than HVHL pitches. Mean elbow varus moment was 110.46 Nm in HVLL pitches and 136.58 Nm in HVHL pitches, corresponding to a cluster-bootstrap mean difference of −26.12 Nm (95% CI, −39.46 to −14.37; *p* < 0.001). Mean shoulder internal rotation moment was 107.44 Nm in HVLL pitches and 129.11 Nm in HVHL pitches, with a mean difference of −21.67 Nm (95% CI, −33.93 to −11.55; *p* < 0.001). The composite velocity-adjusted loading score was also lower in HVLL pitches (mean difference, −2.16 z units; 95% CI, −2.62 to −1.79; *p* < 0.001). Because elbow varus moment, shoulder internal rotation moment, and the composite adjusted loading score contributed to profile definition, these loading differences were treated as profile verification rather than independent biomechanical findings. Profile-verification variables are summarized in Table 3.

The main non-definitional differences were concentrated in lead-knee mechanics. HVLL pitches showed smaller lead-knee extension change from foot plant to ball release than HVHL pitches (6.61° vs. 19.85°; mean difference, −13.25°; 95% CI, −20.37 to −5.58; cluster-bootstrap *p* < 0.001; FDR-adjusted *p* = 0.010). Lead-knee extension angular velocity at foot plant was also lower in HVLL pitches (30.67°/s vs. 232.77°/s; mean difference, −202.10°/s; 95% CI, −293.22 to −88.82; cluster-bootstrap *p* < 0.001; FDR-adjusted *p* = 0.010). Trunk axial rotation at maximum external rotation was lower in HVLL pitches than in HVHL pitches in the cluster-bootstrap analysis (100.35° vs. 109.54°; mean difference, −9.19°; 95% CI, −16.90 to −1.48; cluster-bootstrap *p* = 0.021), but this difference did not remain below the false-discovery-rate threshold (FDR-adjusted *p* = 0.105).

Stride length and maximum horizontal centre-of-gravity velocity showed directional differences but weaker support. HVLL pitches showed directionally greater maximum horizontal centre-of-gravity velocity than HVHL pitches (3.12 vs. 2.93 m/s; mean difference, 0.182 m/s; 95% CI, −0.043 to 0.370; cluster-bootstrap *p* = 0.106; FDR-adjusted *p* = 0.264). Stride length was also directionally greater in HVLL pitches (0.857 vs. 0.822 body heights; mean difference, 0.035 body heights; 95% CI, −0.012 to 0.079; cluster-bootstrap *p* = 0.141; FDR-adjusted *p* = 0.309). Because the confidence intervals crossed zero and the FDR-adjusted *p*-values were not below 0.05, these variables were interpreted as directional tendencies rather than stable between-profile differences. Selected non-definitional biomechanical differences emphasized in the main text are summarized in Table 4, whereas the complete core kinematic comparison family is reported in Supplementary Table S6.

Differences were calculated as HVLL minus HVHL. Confidence intervals and *p*-values were estimated using pitcher-level cluster bootstrap. Pitch speed was used to verify performance comparability between high-velocity profiles. Elbow varus moment, shoulder internal rotation moment, and composite adjusted loading contributed to profile construction and were therefore treated as profile-verification variables rather than independent biomechanical findings. HVLL, high-velocity low-adjusted-load; HVHL, high-velocity high-adjusted-load.

Differences were calculated as HVLL minus HVHL. Confidence intervals and *p*-values were estimated using pitcher-level cluster bootstrap. These variables were not used to define profile membership and were therefore treated as non-definitional biomechanical outcomes. FDR-adjusted *p*-values were calculated using the Benjamini–Hochberg procedure across the complete available non-definitional core kinematic comparison family, not only across the variables displayed in this table. Table 4 displays selected non-definitional variables emphasized in the main text. The complete core kinematic comparison family and corresponding false-discovery-rate-adjusted results are reported in Supplementary Table S6. FP, foot plant; BR, ball release; MER, maximum external rotation; COG, centre of gravity; FDR, false discovery rate; HVLL, high-velocity low-adjusted-load; HVHL, high-velocity high-adjusted-load.

**Figure 4:**
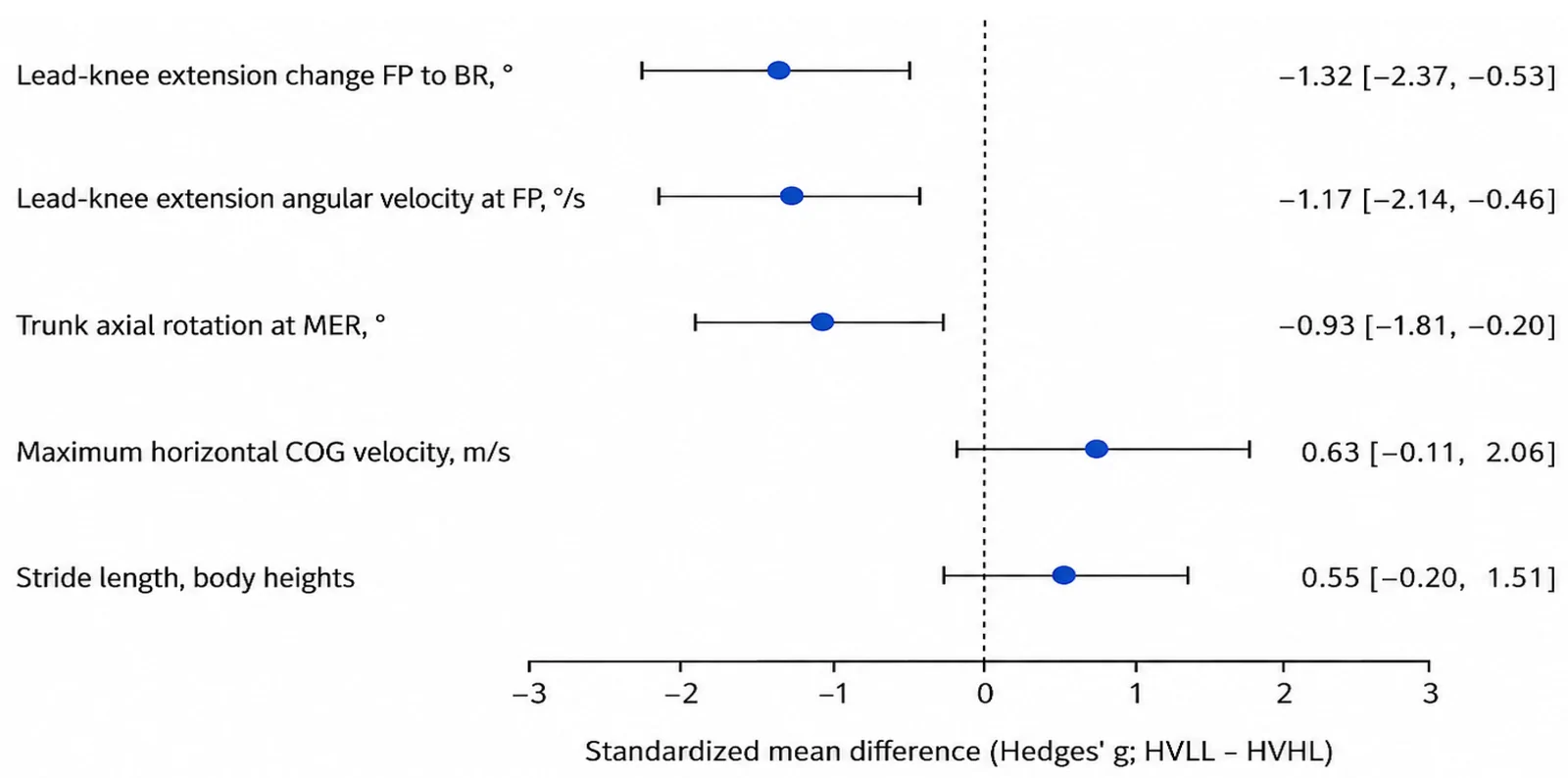
Cluster-bootstrap standardized mean differences.

The selected non-definitional variables were visualized using cluster-bootstrap standardized mean differences in Figure 4 because the variables were measured in different physical units. Lead-knee extension change showed a large negative standardized difference in HVLL relative to HVHL pitches (Hedges’ g = −1.32; 95% CI, −2.37 to −0.53). Lead-knee extension angular velocity at foot plant also showed a large negative standardized difference (Hedges’ g = −1.17; 95% CI, −2.14 to −0.46). Trunk axial rotation at maximum external rotation showed a moderate-to-large negative standardized difference (Hedges’ g = −0.93; 95% CI, −1.81 to −0.20). In contrast, maximum horizontal centre-of-gravity velocity (Hedges’ g = 0.63; 95% CI, −0.11 to 2.06) and stride length (Hedges’ g = 0.55; 95% CI, −0.20 to 1.51) showed positive but uncertain standardized differences because their confidence intervals crossed zero.

Standardized mean differences between HVLL and HVHL pitches are shown for selected non-definitional biomechanical variables. Standardized differences are presented as Hedges’ g values and were calculated as HVLL minus HVHL. Negative values indicate lower values in HVLL pitches. Points represent standardized mean differences, and horizontal lines represent 95% pitcher-level cluster-bootstrap confidence intervals. This figure visualizes effect sizes and confidence intervals; false-discovery-rate-adjusted *p*-values for the selected variables are reported in Table 4, whereas the complete available non-definitional core kinematic comparison family is reported in Supplementary Table S6. Abbreviations are defined as follows: FP, foot plant; BR, ball release; MER, maximum external rotation; COG, centre of gravity; HVLL, high-velocity low-adjusted-load; and HVHL, high-velocity high-adjusted-load.

### 3.4. Sensitivity Analyses

Sensitivity analyses were used to evaluate whether the main findings were influenced by repeated pitches, pitcher-level clustering, cut-off choice, or residual-model specification. Pitcher-level aggregation generally supported the primary cluster-bootstrap findings for lead-knee variables. After averaging variables within each pitcher and profile, HVLL remained associated with lower lead-knee extension change (mean difference, −12.00°; 95% CI, −19.08 to −4.91; *p* = 0.0016) and lower lead-knee extension angular velocity at foot plant (mean difference, −155.07°/s; 95% CI, −279.17 to −30.98; *p* = 0.0159). Trunk axial rotation at maximum external rotation also remained lower in the pitcher-level aggregated comparison (mean difference, −6.94°; 95% CI, −13.11 to −0.77; *p* = 0.0289).

Mixed-effects sensitivity models provided the strongest support for the lead-knee findings. After including pitcher as a random intercept and adjusting for pitch velocity, body mass, body height, age, throwing side, and playing level, the HVLL profile remained associated with lower lead-knee extension change (estimate, −4.56°; 95% CI, −8.72 to −0.39; *p* = 0.032) and lower lead-knee extension angular velocity at foot plant (estimate, −103.15°/s; 95% CI, −199.35 to −6.94; *p* = 0.036). In contrast, the association with trunk axial rotation at maximum external rotation was attenuated in the mixed-effects model (estimate, −1.58°; 95% CI, −4.20 to 1.04; *p* = 0.238). Stride length and maximum horizontal centre-of-gravity velocity were also not clearly supported in mixed-effects sensitivity models.

Cut-off sensitivity analyses showed that lead-knee extension change was the most consistent non-definitional finding across tertile-, quartile-, and median-based profile definitions. Lead-knee extension angular velocity at foot plant showed consistent direction and strong support in the tertile and quartile definitions, with weaker support in the median split. Trunk axial rotation at maximum external rotation was supported in the tertile and median definitions but not in the quartile definition. Stride length and maximum horizontal centre-of-gravity velocity were not consistently supported across cut-off strategies.

A PCA-based loading-score sensitivity analysis supported the equal-weight composite approach. The first principal component of the standardized elbow and shoulder residuals explained 98.05% of the residual-loading variance. The PCA-based loading score was perfectly correlated with the primary equal-weight composite adjusted loading score (r = 1.000 to three decimals), and the first-component loadings were equal for the elbow and shoulder residuals (0.707 and 0.707, respectively). When the high-velocity loading profiles were reconstructed using the PCA-based score, profile assignments were identical to the primary tertile-based classifications. The PCA-based analysis yielded the same HVLL and HVHL sample sizes, complete profile overlap with the primary classification, and directionally identical key non-definitional biomechanical differences. Complete PCA-based sensitivity outputs are provided in Supplementary Table S13 and the Zenodo reproducibility package.

Residual-model sensitivity analyses further clarified the interpretation of the profile differences. When profiles were reconstructed using the fixed-effect residual model, the original pattern of lower lead-knee extension change, lower lead-knee extension angular velocity at foot plant, and lower trunk axial rotation at maximum external rotation was retained. However, when pitcher fixed effects were included in the residual-loading model, the non-definitional lead-knee and trunk differences were substantially attenuated. Under the pitcher fixed-effect residual definition, the HVLL and HVHL profiles each included 50 pitches from 33 pitchers, with 31 pitchers overlapping between profiles, and the differences in the main non-definitional variables all had confidence intervals crossing zero. Together with the minimal pitcher overlap in the primary profiles, these findings indicate that the primary tertile-based results reflect almost entirely inter-individual, between-pitcher biomechanical organization rather than within-pitcher modifiable mechanics.

Complete sensitivity-analysis outputs are provided in the Zenodo reproducibility package.

Overall, the sensitivity analyses indicated that the main non-definitional differences were most consistent at the between-profile level but were attenuated after accounting for pitcher-specific loading baselines, supporting an inter-individual, between-pitcher interpretation rather than a within-pitcher mechanistic interpretation.

All prespecified mixed-effects models converged. They included 84 high-velocity extreme-profile pitches from 32 pitchers, with 1–5 pitches per pitcher (mean, 2.6). Random-intercept variances were 86.64 for lead-knee extension change, 22,924.63 for lead-knee extension angular velocity at foot plant (expressed in squared angular-velocity units, [degrees/s]^2^), and 60.96 for trunk axial rotation at maximum external rotation. The HVLL effect remained negative for both lead-knee outcomes, whereas the trunk association was attenuated. These diagnostics support use of the models as sensitivity analyses while underscoring the limited effective number of pitcher-level clusters.

### 3.5. Supplementary Multivariable Discrimination

Exploratory multivariable discrimination analyses included 84 high-velocity extreme-profile pitches: 47 HVLL pitches and 37 HVHL pitches from 32 unique pitchers. Group-aware cross-validation was used to prevent pitches from the same pitcher from appearing in both training and test folds. These analyses were interpreted as supplementary descriptions of multivariable profile separation, not as externally validated prediction models.

Using the core kinematic predictor set, the random forest model showed the highest mean AUROC (0.819 ± 0.096), whereas histogram-based gradient boosting showed the highest balanced accuracy (0.755 ± 0.082). L2-regularized logistic regression showed a mean AUROC of 0.773 ± 0.214 and a balanced accuracy of 0.735 ± 0.207. Adding extended kinetic, ground-reaction-force, and energy-transfer variables did not consistently improve discrimination. In the expanded predictor set, mean AUROC values ranged from 0.620 to 0.784 across models, indicating greater instability when the exploratory feature set was expanded.

These findings suggest that non-definitional lower-limb, pelvis, trunk, stride, and centre-of-gravity variables contained multivariable signal that separated the predefined HVLL and HVHL profiles. However, because the analysis was based on a small number of high-velocity extreme-profile pitches and pitchers, and because no external validation dataset was available, these results should not be interpreted as evidence of deployable prediction, clinical screening, injury-risk classification, or coaching decision support. Cross-validated AUROC results and permutation-importance rankings are shown in Supplementary Figures S2 and S3, respectively. Complete fold-level cross-validation outputs, class distributions, model settings, and standardized logistic regression coefficients are provided in the Zenodo reproducibility package.

## 4. Discussion

### 4.1. Principal Findings

The central result is interpretive rather than classificatory. Velocity-adjusted profiles could be constructed reproducibly, but only two pitchers contributed to both HVLL and HVHL. The observed kinetic-chain patterns therefore represent an almost entirely inter-individual comparison of between-pitcher performance–loading organization and do not demonstrate within-pitcher mechanisms.

Among the non-definitional variables, lead-knee measures showed the most consistent support across cluster-bootstrap, pitcher-level aggregation, and mixed-effects analyses. Trunk axial rotation showed weaker, model-dependent support, whereas stride length and centre-of-gravity velocity were directional only. Loading variables themselves were treated as profile verification because they contributed to the profile definition.

This distinction was reinforced when pitcher fixed effects were included in the residual-loading models: profile-associated lead-knee and trunk differences were substantially attenuated. The framework is therefore suited to hypothesis generation about pitcher profiles, not to claims that changing one kinematic variable will move an individual pitcher from HVHL to HVLL.

The main contribution is a transparent framework that separates performance output from relative loading while exposing its assumptions through cluster-aware inference, alternative cut-offs, pitcher-fixed-effect residuals, and PCA sensitivity analysis.

### 4.2. Lead-Knee Mechanics and Velocity-Adjusted Loading Profiles

Lead-knee measures were the clearest non-definitional markers of the two high-velocity profiles. Their consistency across several pitcher-aware analyses identifies them as priorities for further investigation, not as demonstrated within-pitcher mechanisms or validated technical interventions.

This result does not show that less lead-knee extension is generally preferable. Existing studies address lead-leg bracing in relation to velocity and energy transfer, whereas the present comparison contrasted high-velocity profiles composed almost entirely of different pitchers. It therefore describes how inter-individual profiles differ, not whether changing knee extension within a pitcher would alter velocity or upper-extremity loading.

The observed lead-knee differences may motivate future investigation of braking and segmental timing, but the present analysis did not directly measure a within-pitcher braking strategy, continuous segmental timing, or energy-transfer pathways. Pitching is a proximal-to-distal sequence in which ground reaction forces, segmental timing, and energy generation, transfer, and absorption interact across the lower extremities and trunk [3,14,15,34,35,36,37,38]. Because the data comprised processed point-of-interest variables and the profile contrast was almost entirely inter-individual, the lead-knee pattern should be interpreted as a descriptive between-pitcher marker, not evidence of less abrupt braking, altered temporal sequencing, or a more efficient transfer mechanism.

Only two pitchers contributed to both high-velocity profiles; thus, the comparison provided almost no direct within-pitcher contrast. Pitcher-fixed-effect residualization also attenuated the lead-knee differences. A within-pitcher interpretation would require repeated observations of the same pitchers under controlled velocity and workload conditions, preferably with intervention or longitudinal designs.

The findings consequently refine rather than overturn lead-leg bracing literature: lead-knee behaviour may help characterize inter-individual performance–loading organization across pitchers, but it does not establish a universal coaching rule, a load-reducing mechanism, or an injury-risk threshold.

### 4.3. Trunk and Pelvis Mechanics

Trunk and pelvis motion is integral to proximal-to-distal sequencing [6,8,9,14,15]. Trunk axial rotation at maximum external rotation was associated with profile separation in the primary and pitcher-aggregated analyses. Given the almost entirely inter-individual profile contrast, this variable should be regarded as a descriptive between-pitcher correlate of profile membership rather than evidence that trunk orientation modifies upper-extremity loading.

Support was weaker after random-intercept adjustment and pitcher-fixed-effect residualization. The trunk pattern may therefore reflect stable pitcher-specific characteristics or correlated between-pitcher differences; the present data cannot distinguish these possibilities from a modifiable within-pitcher movement strategy.

Trunk rotation should not be converted into an immediate technical target. This analysis cannot determine whether changing trunk orientation within a pitcher would reduce arm loading, preserve velocity, or redistribute demand elsewhere in the kinetic chain.

### 4.4. Stride, Centre-of-Gravity Motion, and Extended Kinetic Variables

Stride length and maximum horizontal centre-of-gravity velocity showed directional patterns but were not consistently supported across pitcher-aware and cut-off sensitivity analyses. They should therefore remain contextual observations.

Ground-reaction-force and energy-transfer variables are biomechanically relevant to the generation and transfer of mechanical energy [34,35,36,37,38], yet adding them did not consistently improve exploratory multivariable discrimination. This result may reflect limited sample size, missingness, predictor correlation, and the inability of event-based variables to represent continuous energy flow. These extended variables should not be presented as robust profile determinants in the current dataset.

### 4.5. Exploratory Multivariable Discrimination

The exploratory multivariable discrimination analyses suggested that non-definitional kinetic-chain variables contained signal that separated the predefined HVLL and HVHL profiles. The strongest descriptive performance was observed with the core kinematic predictor set, whereas adding extended kinetic, ground-reaction-force, and energy-transfer variables did not consistently improve discrimination. This pattern suggests that the main profile separation was largely captured by lower-limb, pelvis, trunk, stride, and centre-of-gravity variables rather than by a broader exploratory feature set.

The multivariable discrimination analyses should be interpreted as supplementary and descriptive. Although group-stratified cross-validation was used to reduce pitcher-level leakage, the analysis included only 84 high-velocity extreme-profile pitches from 32 unique pitchers, and the effective number of independent test units in each fold was small. Therefore, the reported AUROC and balanced accuracy values do not establish predictive validity, clinical utility, deployment readiness, or external generalizability. Their role in the present study was limited to describing whether non-definitional kinetic-chain variables contained combined signal separating the predefined profiles. These metrics describe inter-individual profile separation and do not identify biomechanical mechanisms.

The machine-learning results were therefore secondary to the main biomechanical comparisons. Their value was to provide a supplementary multivariable check that the predefined profiles were not separated only by isolated univariate differences. However, the primary evidence for profile-associated biomechanical features remains the pitcher-level cluster-bootstrap, pitcher-level aggregation, mixed-effects sensitivity models, cut-off sensitivity analyses, residual-model sensitivity analyses, and PCA-based loading-score sensitivity analysis. Future work with larger datasets, external validation cohorts, and prospectively defined modelling pipelines would be required before multivariable models based on this framework could be interpreted as predictive tools.

### 4.6. Interpretation of the Velocity-Adjusted Loading Framework

The residual framework estimates whether elbow and shoulder moments are higher or lower than expected for measured performance and athlete characteristics. It improves on raw moments or single-denominator ratios by allowing several covariates to enter simultaneously, but it remains model-dependent and descriptive.

The equal-weight score was chosen a priori for transparency: both residuals were standardized, neither joint was prioritized, and no sample-specific weighting needed to be estimated. PCA was deliberately used as a sensitivity analysis. Its equal loadings, 98.05% explained variance, perfect correlation with the primary score, and identical profile assignments show that the simple average did not materially differ from the data-driven alternative in this sample.

Because the residual components were highly correlated, the composite should be understood as a common adjusted upper-extremity loading index rather than separate elbow- and shoulder-specific mechanisms. It is not an injury-risk score, tissue-tolerance measure, clinical threshold, or athlete-classification tool.

The framework also retained between-pitcher variation. The limited pitcher overlap and attenuation after pitcher-fixed-effect residualization are therefore not peripheral limitations; they define the proper interpretation of the study. The profiles identify groups of pitchers with different performance–loading organization; they do not represent alternative within-pitcher states or movement strategies proven to reduce loading within an individual.

External datasets, larger samples, and within-pitcher repeated-measures studies are required before the framework can be considered stable, generalizable, or practically modifiable.

### 4.7. Applied Implications and Limitations

The applied value of the study is limited to profiling and hypothesis generation. It offers a way to describe relative loading at comparable velocities, but the dataset lacks longitudinal injury surveillance, workload, fatigue, pain, clinical outcomes, and intervention data. HVLL should not be labelled protective, HVHL should not be labelled risky, and the lead-knee or trunk patterns should not be prescribed as isolated coaching changes.

Additional limitations include reliance on processed point-of-interest variables rather than full waveforms, sample-derived thresholds, a small number of extreme-profile pitchers, and no external validation of the supplementary classifiers. Most importantly, the HVLL-versus-HVHL comparison was almost entirely inter-individual, and the profile differences weakened after pitcher-specific loading baselines were modelled.

Future evaluation requires independent datasets, repeated sessions, full time-series biomechanics, and longitudinal workload and health outcomes before velocity-adjusted profiles can be treated as stable or modifiable assessment targets.

### 4.8. Future Directions

Future research should test the velocity-adjusted performance–loading framework in independent baseball pitching datasets and in larger samples with repeated pitches per pitcher. Within-pitcher designs would be particularly valuable because they could determine whether the lead-knee and trunk differences observed here reflect modifiable pitch-to-pitch mechanics or stable pitcher-level movement organization.

Future studies should also examine full time-series biomechanical data rather than only point-of-interest metrics. Waveform-based analyses could clarify whether differences in lead-knee and trunk mechanics emerge before foot plant, during lead-leg braking, near maximum external rotation, or during the acceleration phase. Combining point-of-interest and waveform approaches may provide a more complete view of how lower-limb and trunk mechanics relate to velocity-adjusted upper-extremity loading.

Finally, longitudinal studies that include workload, fatigue, pain, and injury outcomes are needed before performance–loading profiles can be connected to clinical risk or intervention planning. The present study provides a reproducible framework and hypothesis-generating findings, but it does not establish injury prediction or causal technical recommendations.

## 5. Conclusions

This cross-sectional secondary analysis constructed velocity-adjusted elbow–shoulder loading profiles among high-velocity fastballs. Lead-knee measures were the most consistent non-definitional profile markers, whereas trunk axial rotation showed weaker, model-dependent support.

Because only two pitchers contributed to both HVLL and HVHL and the differences attenuated after pitcher-fixed-effect residualization, the comparison was almost entirely inter-individual and should be interpreted as exploratory between-pitcher profile associations. It does not establish within-pitcher mechanisms, injury-risk thresholds, coaching prescriptions, or validated technique targets. Independent datasets and within-pitcher studies are needed to determine stability, generalizability, and modifiability.

## Figures and Tables

**Table 1:** Analytical sample characteristics.

Variable	N	Mean ± SD/n (%)
Pitch speed, km/h	411	136.31 ± 7.60
Elbow varus moment, Nm	411	111.27 ± 19.65
Shoulder internal rotation moment, Nm	411	106.12 ± 18.72
Body mass, kg	411	90.29 ± 9.90
Body height, m	411	1.85 ± 0.07
Age, years	411	21.29 ± 2.25
Stride length, body heights	411	0.84 ± 0.06
Maximum trunk axial rotational velocity, °/s	411	1054.80 ± 88.36
Maximum pelvis axial rotational velocity, °/s	411	751.54 ± 93.67
Maximum hip–shoulder separation, °	411	32.25 ± 6.62
Throwing side: right	328	79.8%
Throwing side: left	83	20.2%
Playing level: college	314	76.4%
Playing level: independent	42	10.2%
Playing level: high school	32	7.8%
Playing level: MiLB	23	5.6%
Pitch type: fastball	411	100.0%

Values are shown as mean ± standard deviation for continuous variables and counts with percentages for categorical variables. MiLB, Minor League Baseball.

**Table 2:** Sample-derived performance–loading profile definitions and sample sizes.

Profile	Definition	n Pitches	n Pitchers	Percentage
HVLL	High velocity and low adjusted load	47	17	11.4%
HVHL	High velocity and high adjusted load	37	17	9.0%
LVLL	Low velocity and low adjusted load	48	16	11.7%
LVHL	Low velocity and high adjusted load	50	15	12.2%
Other	Not classified into an extreme profile	229	79	55.7%

**Table 3:** Profile verification variables between HVLL and HVHL fastball pitches.

Variable	HVLL Mean	HVHL Mean	Difference	95% CI	*p*-Value
Pitch speed, km/h	144.41	143.39	1.01	−0.97 to 2.88	0.310
Elbow varus moment, Nm	110.46	136.58	−26.12	−39.46 to −14.37	<0.001
Shoulder internal rotation moment, Nm	107.44	129.11	−21.67	−33.93 to −11.55	<0.001
Composite adjusted loading, z	−0.98	1.18	−2.16	−2.62 to −1.79	<0.001

**Table 4:** Selected non-definitional biomechanical differences between HVLL and HVHL fastball pitches.

Variable	HVLL	HVHL	Difference	95% CI	*p*-Value	FDR *p*
Lead-knee extension change FP to BR, °	6.61	19.85	−13.25	−20.37 to −5.58	<0.001	0.010
Lead-knee extension angular velocity at FP, °/s	30.67	232.77	−202.10	−293.22 to −88.82	<0.001	0.010
Trunk axial rotation at MER, °	100.35	109.54	−9.19	−16.90 to −1.48	0.021	0.105
Maximum horizontal COG velocity, m/s	3.12	2.93	0.182	−0.043 to 0.370	0.106	0.264
Stride length, body heights	0.857	0.822	0.035	−0.012 to 0.079	0.141	0.309

## Data Availability

The original baseball pitching data analyzed in this study are publicly available from the OpenBiomechanics Project. The original OpenBiomechanics raw data are not redistributed in the present repository. Analysis scripts, source-file verification code, derived datasets, velocity-adjusted loading scores, performance–loading profile classifications, statistical outputs, sensitivity analyses, machine-learning outputs, supplementary tables, figures, figure-generation files, pinned software-environment files, and source-version records are available in the Zenodo reproducibility package at https://doi.org/10.5281/zenodo.21620946.

## Supplementary Materials

The following supporting information can be downloaded at: https://www.mdpi.com/article/10.3390/sports14080338/s1. Table S1: Variable dictionary; Table S2: Sample-screening flow; Table S3: Missing-data summary; Table S4: Residual-model outputs and diagnostics; Table S5: Complete manuscript-facing between-profile comparisons; Table S6: Core kinematic comparison family and FDR results; Table S7: Pitcher-level aggregated analyses; Table S8: Mixed-effects sensitivity models; Table S9: Cut-off sensitivity analyses; Table S10: Residual-model sensitivity analyses; Table S11: Machine-learning fold structure and model settings; Table S12: Exploratory multivariable discrimination performance; Table S13: PCA-based adjusted loading sensitivity analysis; Figure S1: Residual-model diagnostic summary; Figure S2: Exploratory cross-validated AUROC; Figure S3: Permutation-importance ranking.
