# Supplementary material for: Exploratory Velocity-Adjusted Upper-Extremity Loading Profiles in Fastball Pitching: A Secondary Analysis of OpenBiomechanics Data
Source: Sports (Basel). 2026 Aug 5;14(8):338. doi: 10.3390/sports14080338 (PMC13517245; doi:10.3390/sports14080338)
Supplement: Supplementary file 1 [file sports-14-00338-s001.zip › sports-4440188-supplementary.pdf]

# Supplementary Materials

## Exploratory Velocity-Adjusted Upper-Extremity Loading Profiles in Fastball Pitching: A Secondary Analysis of OpenBiomechanics Data

Yipu Zhang, Yue Wu, and Han-Kyo Seo*

Department of Sports Medicine, Shinhan University, Uijeongbu-si 11644, Republic of Korea

* Correspondence: hkseo@shinhan.ac.kr

Associated reproducibility package: https://doi.org/10.5281/zenodo.21620946

Source snapshot: OpenBiomechanics repository commit 8a3e889533d095a9247d70be44a861130201049a (verified 30 June 2026)

**Scope and provenance.** This consolidated file reports the supplementary tables and figures cited in the production proof. It reproduces exact statistics available in the current proof and validated result files. Complete machine-readable row-level outputs, cross-validation split files, analysis scripts, and source-verification records remain archived in the Zenodo package.

## Contents

**Table S1:** Variable dictionary
**Table S2:** Sample-screening flow
**Table S3:** Missing-data summary
**Table S4:** Residual-model outputs and diagnostics
**Table S5:** Complete manuscript-facing between-profile comparisons
**Table S6:** Core kinematic comparison family and FDR results
**Table S7:** Pitcher-level aggregated analyses
**Table S8:** Mixed-effects sensitivity models
**Table S9:** Cut-off sensitivity analyses
**Table S10:** Residual-model sensitivity analyses
**Table S11:** Machine-learning fold structure and model settings
**Table S12:** Exploratory multivariable discrimination performance
**Table S13:** PCA-based adjusted loading sensitivity analysis
**Figure S1:** Residual-model diagnostic summary
**Figure S2:** Exploratory cross-validated AUROC
**Figure S3:** Permutation-importance ranking

***Note:*** *Abbreviations: AUROC, area under the receiver operating characteristic curve; BR, ball release; CI, confidence interval; COG, centre of gravity; FDR, false discovery rate; FP, foot plant; GRF, ground-reaction force; HVHL, high-velocity high-adjusted-load; HVLL, high-velocity low-adjusted-load; MER, maximum external rotation; MiLB, Minor League Baseball; PCA, principal component analysis; RMSE, root mean square error; VIF, variance inflation factor.*

## Supplementary Tables

### Table S1. Variable dictionary

| Dataset field / derived variable | Manuscript label | Definition | Unit | Analytical role |
|---|---|---|---|---|
| session_pitch | Linkage identifier | Pitch-level key used to merge metadata.csv and poi_metrics.csv. | Identifier | Not analysed as a predictor |
| Pitcher identifier | Clustering identifier | Anonymized athlete identifier used for cluster bootstrap, aggregation, grouped cross-validation, and random intercepts. | Identifier | Clustering/grouping |
| pitch_speed_mph | Pitch velocity | Fastball velocity in the source data; converted to km/h for manuscript-facing output. | mph; km/h | Performance/profile definition |
| session_mass_kg | Body mass | Athlete body mass recorded for the session. | kg | Residual-model covariate |
| session_height_m | Body height | Athlete body height recorded for the session. | m | Residual-model covariate |
| age_yrs | Age | Athlete age at data collection. | years | Residual-model covariate |
| p_throws_R | Throwing side | Indicator for right-handed throwing; left-handed throwing was the reference category. | binary | Residual-model covariate |
| playing_level_* | Playing level | Indicators for high school, independent, and MiLB; college was the reference category. | categorical | Residual-model covariate |
| elbow_varus_moment | Elbow varus moment | Pitch-level upper-extremity loading indicator. | Nm | Profile-defining loading outcome |
| shoulder_internal_rotation_moment | Shoulder internal rotation moment | Pitch-level upper-extremity loading indicator. | Nm | Profile-defining loading outcome |
| Elbow standardized residual | Adjusted elbow loading | Standardized residual from the elbow varus moment adjustment model. | z | Composite component |
| Shoulder standardized residual | Adjusted shoulder loading | Standardized residual from the shoulder internal rotation moment adjustment model. | z | Composite component |
| Composite adjusted loading | Velocity-adjusted loading score | Equal-weight mean of standardized elbow and shoulder residuals. | z | Profile definition |
| stride_length | Stride length | Stride distance normalized to body height. | body heights | Core kinematic outcome |
| stride_angle | Stride angle | Direction of stride relative to the source coordinate definition. | degrees | Core kinematic outcome |
| lead_knee_extension_angular_velo_fp | Lead-knee extension angular velocity at FP | Lead-knee angular velocity at foot plant. | degrees/s | Core kinematic outcome |
| lead_knee_extension_from_fp_to_br | Lead-knee extension change FP to BR | Change in lead-knee extension angle from foot plant to ball release. | degrees | Core kinematic outcome |
| max_trunk_rotational_velo | Maximum trunk axial rotational velocity | Peak trunk axial rotational velocity. | degrees/s | Core kinematic outcome |
| max_pelvis_rotational_velo | Maximum pelvis axial rotational velocity | Peak pelvis axial rotational velocity. | degrees/s | Core kinematic outcome |
| max_hip_shoulder_separation | Maximum hip–shoulder separation | Maximum pelvis-to-trunk rotational separation. | degrees | Core kinematic outcome |
| torso_anterior_tilt_* | Trunk anterior tilt | Trunk anterior tilt at specified pitching events (FP, MER, or BR). | degrees | Core kinematic outcome |
| torso_lateral_tilt_* | Trunk lateral tilt | Trunk lateral tilt at specified pitching events. | degrees | Core kinematic outcome |
| torso_rotation_* | Trunk axial rotation | Trunk axial rotation at specified pitching events; torso_rotation_mer was a selected main outcome. | degrees | Core kinematic outcome |
| pelvis_anterior_tilt_* | Pelvis anterior tilt | Pelvis anterior tilt at specified pitching events. | degrees | Core kinematic outcome |
| pelvis_lateral_tilt_* | Pelvis lateral tilt | Pelvis lateral tilt at specified pitching events. | degrees | Core kinematic outcome |
| pelvis_rotation_* | Pelvis axial rotation | Pelvis axial rotation at specified pitching events. | degrees | Core kinematic outcome |

| Dataset field / derived variable | Manuscript label | Definition | Unit | Analytical role |
|---|---|---|---|---|
| max_cog_velo_x | Maximum horizontal COG velocity | Maximum horizontal centre-of-gravity velocity. | m/s | Core kinematic outcome |
| COG velocity at peak knee height | Horizontal COG velocity at PKH | Horizontal centre-of-gravity velocity at peak knee height. | m/s | Core kinematic outcome |
| Lead/rear GRF variables | Ground-reaction-force metrics | Lead- and rear-leg force variables used in exploratory expanded models. | N | Extended exploratory predictor |
| Energy-transfer variables | Segmental energy transfer | Hip, knee, pelvis–lumbar, and distal thorax energy-transfer metrics. | J | Extended exploratory predictor |
| Energy generation/absorption | Segmental work-related metrics | Exploratory energy generation and absorption variables. | J | Extended exploratory predictor |

***Note:*** *Asterisks denote event-specific variables. FP, foot plant; MER, maximum external rotation; BR, ball release; PKH, peak knee height. Exact released field names and direction conventions are preserved in the machine-readable variable dictionary in the reproducibility package.*

### Table S2. Sample-screening flow

| Screening stage | Retained sample | Decision / interpretation |
|---|---|---|
| metadata.csv records | 411 | Pitch/session descriptors |
| poi_metrics.csv records | 411 | Point-of-interest biomechanical metrics |
| Merged records | 411 | No duplicate session_pitch records retained |
| Fastball records | 411 | All included records coded as fastballs |
| Unique pitchers | 100 | Independent clustering unit |
| Primary residual-model complete cases | 411 | All loading outcomes and adjustment variables complete |
| HVLL profile | 47 pitches / 17 pitchers | High velocity and low adjusted load |
| HVHL profile | 37 pitches / 17 pitchers | High velocity and high adjusted load |
| Pitchers represented in both HVLL and HVHL | 2 | Primary comparison was almost entirely inter-individual |
| LVLL profile | 48 pitches / 16 pitchers | Low velocity and low adjusted load |
| LVHL profile | 50 pitches / 15 pitchers | Low velocity and high adjusted load |
| Not assigned to an extreme profile | 229 pitches | Middle-tertile or discordant observations |

***Note:*** *High velocity was defined as ≥ 140.33 km/h and low velocity as ≤ 133.41 km/h. Low adjusted loading was defined as ≤ −0.447 and high adjusted loading as ≥ 0.355.*

### Table S3. Missing-data summary

| Variable family | Availability | Handling |
|---|---|---|
| Pitch velocity and adjustment covariates | 411/411 complete | Complete-case residual models used all pitches |
| Elbow varus moment | 411/411 complete | No imputation |
| Shoulder internal rotation moment | 411/411 complete | No imputation |
| Core kinematic comparison family | Complete for the primary analytical sample | No imputation for main biomechanical comparisons |
| Extended kinetic, GRF, and energy-transfer variables | Small amount of missingness (approximately 1.9% overall) | Complete-case analysis within each exploratory model/output |
| peak_rfd_rear and peak_rfd_lead | Excluded after screening | Extreme/unstable values; not used in the main analysis |

***Note:*** *No values were imputed. Exact field-level missing counts and complete-case sample sizes are retained in the archived machine-readable missing-data output.*

**Table S4. Residual-model outputs and diagnostics**

**Table S4A. Model-level fit summary**

| Outcome | n | $R^2$ | Adjusted $R^2$ | RMSE (Nm) | MAE (Nm) | Mean actual (Nm) | SD actual (Nm) | Residual SD (Nm) |
|---|---|---|---|---|---|---|---|---|
| Elbow varus moment | 411 | 0.512 | 0.502 | 13.71 | 10.80 | 111.27 | 19.65 | 13.72 |
| Shoulder internal rotation moment | 411 | 0.559 | 0.550 | 12.41 | 9.77 | 106.12 | 18.72 | 12.43 |

**Table S4B. Residual-model coefficients**

| Model term | Elbow varus moment coefficient | Shoulder internal rotation moment coefficient |
|---|---|---|
| Intercept | −59.0621 | −83.7829 |
| Pitch speed, mph | 1.3195 | 1.3745 |
| Body mass, kg | 1.1231 | 1.0251 |
| Body height, m | −9.2702 | −1.3781 |
| Age, years | −1.2316 | −0.8387 |
| Right-handed throwing indicator | −0.1200 | 1.0953 |
| High school indicator | −3.9032 | −4.6057 |
| Independent indicator | 1.4711 | 0.7239 |
| MiLB indicator | 14.3234 | 13.3050 |

***Note:*** *Model coefficients use the source pitch-speed unit (mph) for exact reproducibility. Left-handed throwing and college playing level were the omitted reference categories.*

**Table S4C. Variance inflation factors**

| Predictor | VIF (identical in both models) |
|---|---|
| Pitch speed | 1.433 |
| Body mass | 1.704 |
| Body height | 1.463 |
| Age | 2.938 |
| Right-handed throwing | 1.091 |
| High school | 1.324 |
| Independent | 2.012 |
| MiLB | 1.541 |

***Note:*** *Both models used the same complete predictor matrix. The VIF range was 1.09–2.94, with no evidence of substantial multicollinearity.*

**Table S5. Complete manuscript-facing between-profile comparisons**

| Variable | HVLL | HVHL | Difference | 95% cluster-bootstrap CI | p value | Hedges' g | FDR p |
|---|---|---|---|---|---|---|---|

| Variable | HVLL | HVHL | Difference | 95% cluster-bootstrap CI | p value | Hedges' g | FDR p |
|---|---|---|---|---|---|---|---|
| Pitch speed, km/h | 144.41 | 143.39 | 1.01 | −0.97 to 2.88 | 0.310 | 0.358 | — |
| Elbow varus moment, Nm | 110.46 | 136.58 | −26.12 | −39.46 to −14.37 | <0.001 | −1.547 | — |
| Shoulder internal rotation moment, Nm | 107.44 | 129.11 | −21.67 | −33.93 to −11.55 | <0.001 | −1.399 | — |
| Composite adjusted loading, z | −0.98 | 1.18 | −2.16 | −2.62 to −1.79 | <0.001 | −3.436 | — |
| Lead-knee extension change FP to BR, ° | 6.61 | 19.85 | −13.25 | −20.37 to −5.58 | <0.001 | −1.320 | 0.010 |
| Lead-knee extension angular velocity at FP, °/s | 30.67 | 232.77 | −202.10 | −293.22 to −88.82 | <0.001 | −1.173 | 0.010 |
| Trunk axial rotation at MER, ° | 100.35 | 109.54 | −9.19 | −16.90 to −1.48 | 0.021 | −0.932 | 0.105 |
| Maximum horizontal COG velocity, m/s | 3.12 | 2.93 | 0.182 | −0.043 to 0.370 | 0.106 | 0.627 | 0.264 |
| Stride length, body heights | 0.857 | 0.822 | 0.035 | −0.012 to 0.079 | 0.141 | 0.547 | 0.309 |

***Note:*** *Differences were calculated as HVLL minus HVHL. Elbow, shoulder, and composite-loading rows are profile-verification variables. FDR values apply to the prespecified non-definitional core kinematic comparison family.*

**Table S6. Complete core kinematic comparison family and FDR results**

| Prespecified core variable/family | Reporting status | Difference | 95% CI | p | FDR p | Interpretation |
|---|---|---|---|---|---|---|
| Stride length | Selected | 0.035 | −0.012 to 0.079 | 0.141 | 0.309 | Directional; CI crossed zero |
| Stride angle | Family member | — | — | — | — | Not emphasized; full numeric row archived |
| Lead-knee extension angular velocity at FP | Selected | −202.10 | −293.22 to −88.82 | <0.001 | 0.010 | FDR-supported |
| Lead-knee extension change FP to BR | Selected | −13.25 | −20.37 to −5.58 | <0.001 | 0.010 | FDR-supported |
| Maximum trunk axial rotational velocity | Family member | — | — | — | — | Not emphasized; full numeric row archived |
| Maximum pelvis axial rotational velocity | Family member | — | — | — | — | Not emphasized; full numeric row archived |
| Maximum hip–shoulder separation | Family member | — | — | — | — | Not emphasized; full numeric row archived |
| Trunk anterior tilt (prespecified events) | Family member | — | — | — | — | Not emphasized; full numeric rows archived |
| Trunk lateral tilt (prespecified events) | Family member | — | — | — | — | Not emphasized; full numeric rows archived |
| Trunk axial rotation (prespecified events) | Selected at MER | −9.19 | −16.90 to −1.48 | 0.021 | 0.105 | Nominal difference; not FDR-supported |
| Pelvis anterior tilt (prespecified events) | Family member | — | — | — | — | Not emphasized; full numeric rows archived |
| Pelvis lateral tilt (prespecified events) | Family member | — | — | — | — | Not emphasized; full numeric rows archived |
| Pelvis axial rotation (prespecified events) | Family member | — | — | — | — | Not emphasized; full numeric rows archived |
| Maximum horizontal COG velocity | Selected | 0.182 | −0.043 to 0.370 | 0.106 | 0.264 | Directional; CI crossed zero |
| Horizontal COG velocity at peak knee height | Family member | — | — | — | — | Not emphasized; full numeric row archived |

***Note:*** *The complete machine-readable variable-level table contains all released event-specific rows. Dashes indicate rows not reproduced numerically in the production proof; no values were inferred or invented in this consolidated document.*

**Table S7. Pitcher-level aggregated analyses**

| Outcome | HVLL − HVHL difference | 95% CI | p value | Interpretation |
|---|---|---|---|---|
| Lead-knee extension change FP to BR, ° | −12.00 | −19.08 to −4.91 | 0.0016 | Supported |
| Lead-knee extension angular velocity at FP, °/s | −155.07 | −279.17 to −30.98 | 0.0159 | Supported |
| Trunk axial rotation at MER, ° | −6.94 | −13.11 to −0.77 | 0.0289 | Supported at pitcher-aggregated level |

***Note:*** *Variables were averaged within pitcher and profile before comparison. These analyses reduce the influence of unequal pitch counts per pitcher but remain predominantly between-pitcher contrasts.*

**Table S8. Mixed-effects sensitivity models**

| Outcome | HVLL estimate | 95% CI | p value | Random-intercept variance | Model result |
|---|---|---|---|---|---|
| Lead-knee extension change FP to BR, ° | −4.56 | −8.72 to −0.39 | 0.032 | 86.64 | Converged; negative HVLL effect |
| Lead-knee extension angular velocity at FP, °/s | −103.15 | −199.35 to −6.94 | 0.036 | 22,924.63 | Converged; negative HVLL effect |
| Trunk axial rotation at MER, ° | −1.58 | −4.20 to 1.04 | 0.238 | 60.96 | Converged; association attenuated |

***Note:*** *All models included 84 pitches from 32 pitchers, with 1–5 pitches per pitcher (mean, 2.6). Models adjusted for pitch velocity, body mass, body height, age, throwing side, and playing level. The angular-velocity variance is expressed in squared angular-velocity units, (degrees/s)².*

**Table S9. Cut-off sensitivity analyses**

| Outcome | Tertile definition | Quartile definition | Median definition | Overall interpretation |
|---|---|---|---|---|
| Lead-knee extension change FP to BR | Supported | Supported | Supported | Most consistent non-definitional result |
| Lead-knee extension angular velocity at FP | Strong support | Strong support | Weaker support | Direction retained across definitions |
| Trunk axial rotation at MER | Supported | Not supported | Supported | Cut-off dependent |
| Maximum horizontal COG velocity | Not consistently supported | Not consistently supported | Not consistently supported | Directional only |
| Stride length | Not consistently supported | Not consistently supported | Not consistently supported | Directional only |

***Note:*** *Cut-off sensitivity analyses were robustness checks. Sample-derived cut-offs are not normative, clinical, injury-risk, or coaching thresholds.*

**Table S10. Residual-model sensitivity analyses**

| Residual definition | HVLL pitches / pitchers | HVHL pitches / pitchers | Overlapping pitchers | Non-definitional pattern | Interpretation |
|---|---|---|---|---|---|
| Primary fixed-effect residual model | 47 / 17 | 37 / 17 | 2 | Lower lead-knee change and velocity; lower trunk rotation at MER in primary analysis | Between-profile association with substantial between-pitcher contribution |
| Pitcher fixed-effect residual model | 50 / 33 | 50 / 33 | 31 | All main non-definitional confidence intervals crossed zero | Primary differences substantially attenuated after modelling pitcher-specific loading baselines |

***Note:*** *Together with only two overlapping pitchers in the primary profiles, this sensitivity analysis supports an almost entirely inter-individual interpretation and does not establish within-pitcher modifiable mechanics.*

**Table S11. Machine-learning fold structure and model settings**

**Table S11A. Grouped cross-validation structure**

| Item / fold | Distribution constraint | Verification / location |
| --- | --- | --- |
| Analytical sample | 84 pitches; 47 HVLL and 37 HVHL | 32 unique pitchers |
| Cross-validation | Five-fold stratified group cross-validation | Pitcher ID was the grouping variable |
| Leakage control | No pitcher appeared in both training and test data within a fold | Profile-defining variables excluded from predictors |
| Fold 1 | Pitcher-disjoint and approximately class-stratified | Exact train/test counts archived |
| Fold 2 | Pitcher-disjoint and approximately class-stratified | Exact train/test counts archived |
| Fold 3 | Pitcher-disjoint and approximately class-stratified | Exact train/test counts archived |
| Fold 4 | Pitcher-disjoint and approximately class-stratified | Exact train/test counts archived |
| Fold 5 | Pitcher-disjoint and approximately class-stratified | Exact train/test counts archived |

**Table S11B. Fixed model settings**

| Model / predictor set | Prespecified setting | Application |
| --- | --- | --- |
| L2-regularized logistic regression | C = 1.0; liblinear solver; balanced class weights | Core and expanded predictor sets |
| Random forest | 500 trees; maximum depth = 4; minimum leaf size = 4; balanced class weights | Core and expanded predictor sets |
| Histogram-based gradient boosting | 200 iterations; learning rate = 0.03; maximum leaf nodes = 7; L2 regularization = 0.1 | Core and expanded predictor sets |
| Core predictor set | Lower-limb, pelvis, trunk, stride, and COG variables | No profile-defining variables |
| Expanded predictor set | Core set plus exploratory kinetic, GRF, and energy-transfer variables | Complete-case inputs |

***Note:*** *Pitch velocity, elbow varus moment, shoulder internal rotation moment, both adjusted residuals, the equal-weight composite score, and the PCA score were excluded from machine-learning inputs. Exact fold-level class and pitcher counts are retained in the archived fold-distribution files.*

**Table S12. Exploratory multivariable discrimination performance**

| Predictor set | Model | AUROC, mean ± SD | Balanced accuracy, mean ± SD |
| --- | --- | --- | --- |
| Core kinematics | L2-regularized logistic regression | 0.773 ± 0.214 | 0.735 ± 0.207 |
| Core kinematics | Random forest | 0.819 ± 0.096 | 0.717 ± 0.123 |
| Core kinematics | HistGradientBoosting | 0.802 ± 0.097 | 0.755 ± 0.082 |
| Expanded kinetics + GRF | L2-regularized logistic regression | 0.710 ± 0.301 | 0.735 ± 0.249 |
| Expanded kinetics + GRF | Random forest | 0.784 ± 0.135 | 0.736 ± 0.140 |
| Expanded kinetics + GRF | HistGradientBoosting | 0.620 ± 0.251 | 0.540 ± 0.177 |

***Note:*** *Values summarize five grouped folds. These models describe supplementary multivariable separation of predefined profiles; they are not externally validated prediction, screening, injury-risk, or coaching tools.*

**Table S13. PCA-based adjusted loading sensitivity analysis**

| PCA sensitivity item | Result |
|---|---|
| Elbow–shoulder standardized residual correlation | 0.960986 |
| PC1 explained variance ratio | 0.980493 (98.05%) |
| PC2 explained variance ratio | 0.019507 (1.95%) |
| PC1 loading: elbow residual | 0.707107 |
| PC1 loading: shoulder residual | 0.707107 |
| Correlation of PCA score with equal-weight composite score | 1.000000 |
| Primary high-velocity cut-off | 87.20 mph / 140.33 km/h |
| Primary loading cut-offs | −0.447982 and 0.355655 |
| PCA loading cut-offs | −0.633543 and 0.502972 |
| HVLL/HVHL profile assignments | Identical to primary classification |
| Key non-definitional directions | Identical to the primary equal-weight analysis |

***Note:*** *The equal component loadings and perfect score correlation support interpretation of the composite as a shared upper-extremity loading dimension rather than two independent domains.*

## Supplementary Figures

**Figure S1. Residual-model diagnostic summary**

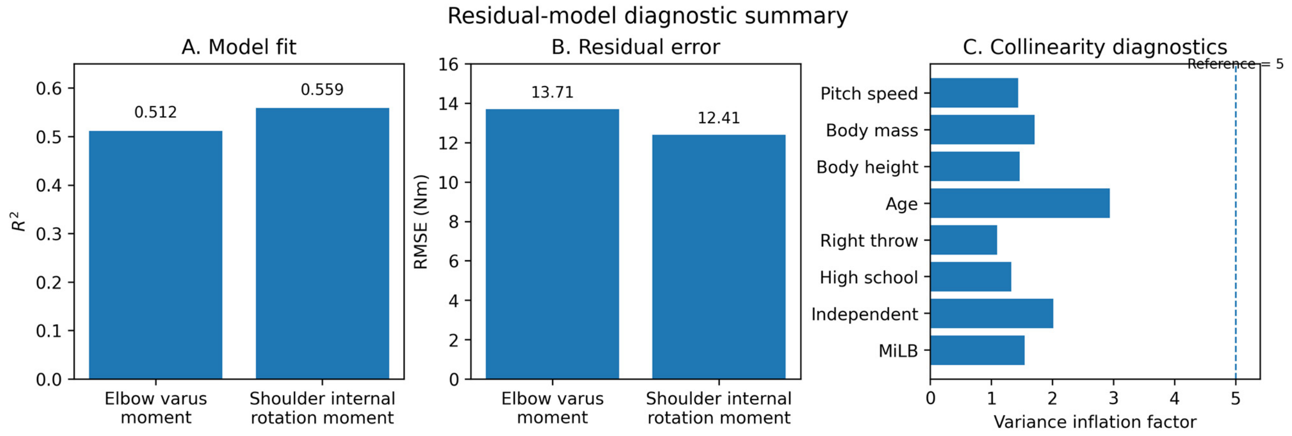


***Note:*** *Panel A reports model $R^2$. Panel B reports RMSE. Panel C reports term-specific VIF values; the dashed reference line is VIF = 5. Both residual models used 411 complete pitches. Standardized elbow and shoulder residuals were highly correlated (r = 0.961).*

**Figure S2. Exploratory cross-validated AUROC**

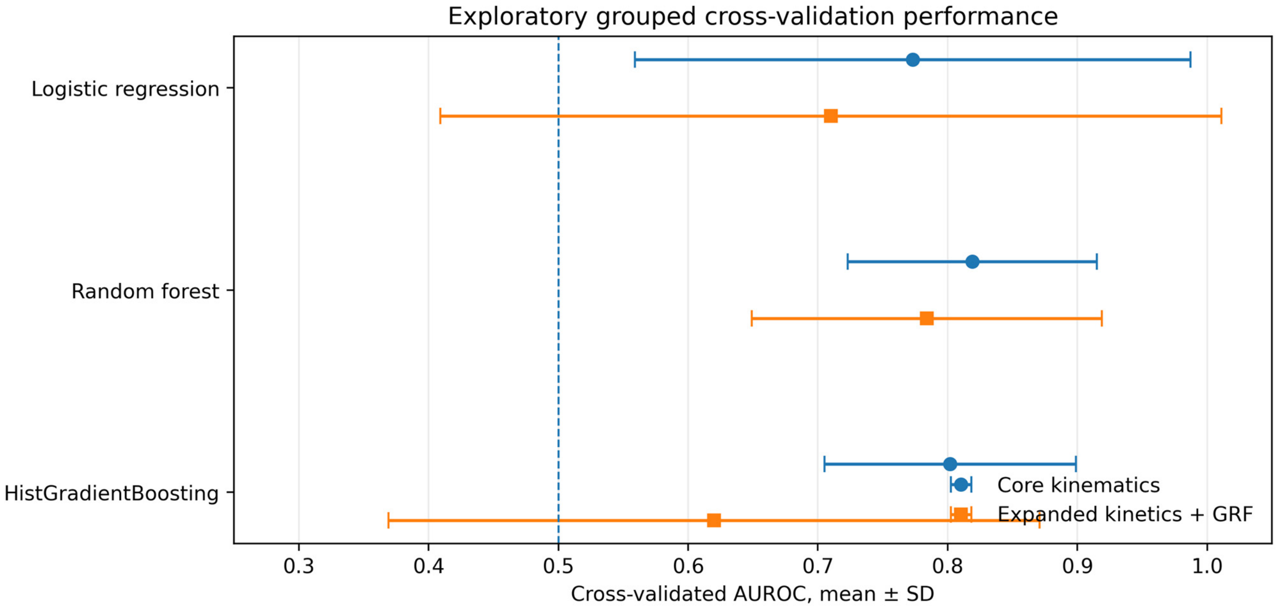


***Note:*** *Points show mean AUROC across five stratified group cross-validation folds and error bars show standard deviations. The vertical dashed line indicates AUROC = 0.5. Pitcher ID was used as the grouping variable. Results are descriptive and do not represent external validation.*

**Figure S3. Permutation-importance ranking**

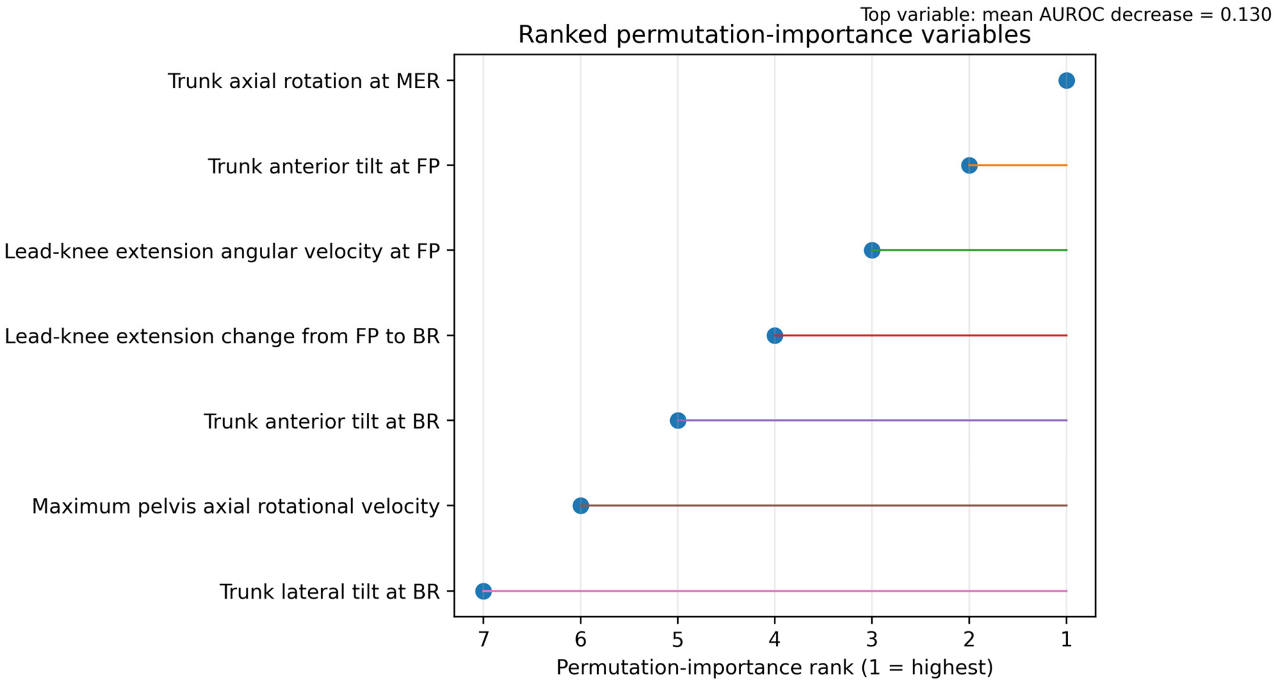


***Note:*** *The figure reports the reproducibly documented ranking of the leading core-kinematic variables. Trunk axial rotation at MER had the highest reported mean AUROC decrease (0.130). Full feature-level magnitudes, repeated-permutation variability, and standardized logistic-regression coefficients are retained in the machine-readable model-interpretation outputs. The ranking is descriptive and must not be interpreted causally.*

## Reproducibility and source records

| Item | Record |
|---|---|
| Zenodo DOI | 10.5281/zenodo.21620946 |
| Package version | 1.1.0 |
| OpenBiomechanics commit | 8a3e889533d095a9247d70be44a861130201049a |
| Verification date | 30 June 2026 |
| metadata.csv SHA-256 | ec5686fef140e728193d4ce4bb16aa116751e716963bb2aedca11111b03816e3 |
| poi_metrics.csv SHA-256 | 5ba2ac8536fba1d6997ebd81d7b1956c73fbb2e141b9c6156cd76f66265f391e |
| Software | Python 3.11.9; NumPy 2.4.6; pandas 3.0.3; SciPy 1.17.1; statsmodels 0.14.6; scikit-learn 1.9.0; matplotlib 3.11.0 |

***Note:*** *The original OpenBiomechanics raw files are not redistributed in the reproducibility package. Source-verification instructions, checksums, analysis scripts, derived datasets, statistical outputs, and figure source data are provided.*
